# Mitochondrial Reshaping Accompanies Neural Differentiation in the Developing Spinal Cord

**DOI:** 10.1371/journal.pone.0128130

**Published:** 2015-05-28

**Authors:** Valérie Mils, Stéphanie Bosch, Julie Roy, Sophie Bel-Vialar, Pascale Belenguer, Fabienne Pituello, Marie-Christine Miquel

**Affiliations:** 1 Universités de Toulouse, Centre de Biologie du Développement, CNRS UMR5547, Université Paul Sabatier, Toulouse, France; 2 UPMC Université Pierre et Marie Curie, Sorbonne Universités, Paris, France; Instituto de Neurociencias, CSIC and UMH, SPAIN

## Abstract

Mitochondria, long known as the cell powerhouses, also regulate redox signaling and arbitrate cell survival. The organelles are now appreciated to exert additional critical roles in cell state transition from a pluripotent to a differentiated state through balancing glycolytic and respiratory metabolism. These metabolic adaptations were recently shown to be concomitant with mitochondrial morphology changes and are thus possibly regulated by contingencies of mitochondrial dynamics. In this context, we examined, for the first time, mitochondrial network plasticity during the transition from proliferating neural progenitors to post-mitotic differentiating neurons. We found that mitochondria underwent morphological reshaping in the developing neural tube of chick and mouse embryos. In the proliferating population, mitochondria in the mitotic cells lying at the apical side were very small and round, while they appeared thick and short in interphase cells. In differentiating neurons, mitochondria were reorganized into a thin, dense network. This reshaping of the mitochondrial network was not specific of a subtype of progenitors or neurons, suggesting that this is a general event accompanying neurogenesis in the spinal cord. Our data shed new light on the various changes occurring in the mitochondrial network during neurogenesis and suggest that mitochondrial dynamics could play a role in the neurogenic process.

## Introduction

Mitochondria are not only key energy-generating organelles that participate in numerous biosynthetic and metabolic pathways, but also crucial regulators of apoptosis [1]. Recent studies in stem cells demonstrated that mitochondria also play critical roles in cell state transition. Using *in vitro* models, it was indeed reported that the transition from a pluripotent to a differentiated state is reversible and partly controlled by the balance between glycolytic and respiratory metabolism [2,3]. Embryonic and adult pluripotent stem cells are characterized by a high activity of glycolytic enzymes accompanied by a low mitochondrial mass and mtDNA content [4,5]. This maintenance of a glycolytic state in progenitors preserves them from oxidative damage and contributes to committed differentiation [2,6]. The later switch to oxidative phosphorylation better fits the high energy demand of differentiating cells [2]. Accordingly, repression of oxidative metabolism and activation of glycolysis are an obligatory steps for adult cells reprograming into iPS [3,7,8]. Of recent interest, mitochondrial morphology changes come with these reversible metabolic adaptations and are thus possibly regulated by contingencies of mitochondrial dynamics [4,9].

The plasticity of the mitochondrial network relies on fission and fusion processes of outer and inner membranes that control mitochondrial morphology. When fission prevails, mitochondria appear as dots, whereas when fusion predominates, mitochondria form a filamentous and interconnected network. Furthermore, this mitochondrial dynamics allows immediate adaptation of the organelles to energetic needs, keeping mitochondria in good health by restoring or removing damaged organelles or precipitating apoptosis in cases of severe defects [10,11].

Mitochondrial dynamics results from the balance between the opposing forces of two distinct machineries: one involving Mitofusins 1 and 2 (MFN1/2) and OPA1 for fusion and the other involving DRP1 for fission. Knocking out DRP1 or MFN1/2 genes, as well as homozygous OPA1 mutations, in mouse models induces midgestation embryonic lethality [12–16], demonstrating the major contribution of mitochondrial dynamics to development. However, while this regulatory process is common to many cell types, the association of OPA1 and MFN2 mutations with severe human neuropathies underlines the singular importance of mitochondrial dynamics in neurons [17,18]. Accordingly, conditional brain-specific DRP1 or MFN2 ablation in mice led to numerous defects in central nervous system development [15,16,19]. Neural cell-specific DRP1^-/-^ mice display a smaller forebrain [15] and a smaller cerebellum, with completely smooth surfaces [16]. Furthermore, targeted deletion of DRP1 induced neurodegeneration in post-mitotic Purkinje cells [20]. Conditional specific MFN2 knockout led to massive post-natal Purkinje cell degeneration [19] and a severe loss of dopaminergic axonal projections in the striatum [21]. Finally, in both primary neurons from these mice models, neuritogenesis and synaptogenesis were impaired as they were in wild-type neurons in which effectors of mitochondrial dynamics were inactivated [15,20,22–25]. Altogether, these data suggest that mitochondrial dynamics plays an essential role in neuronal differentiation and maturation.

Although evidence has begun to accumulate indicating that mitochondrial dynamics play a crucial role in the metabolic adaptations observed during the transition from a multipotent neural progenitor state to a differentiated state [4,9], a clear picture of the mitochondrial morphology changes accompanying the transition from proliferating progenitors to post-mitotic differentiating neurons is still missing. To fill this gap, we analyzed mitochondrial morphology using the paradigm of the developing neural tube of chick and mouse embryos at the prospective spinal cord level. The spinal cord develops from a caudal stem zone containing uncommitted progenitors. Progenitors leaving the stem zone form the neural plate folding into the neural tube. The neural tube presents the advantage of a simple anatomical organization, with proliferating progenitors confined to the ventricular zone and differentiating neurons located at the periphery in the mantle zone. The ventricular zone is a pseudoepithelium where neural progenitors display an elongated shape, with cytoplasmic connections both to the apical and basal surfaces and nuclei performing oscillatory movements in phase with the cell cycle; *i*.*e*., interkinetic nuclear migration [26]. Upon the action of intrinsic and environmental signals, successive waves of neural progenitors are committed to a neuronal fate and exit the cell cycle. After their last mitosis at the apical side, these committed cells migrate out of the ventricular zone toward the mantle zone, where they differentiate into neurons. The molecular networks orchestrating these neurogenesis steps in the developing neural tube are well known [27,28] and numerous markers allow identifying specific populations of neural progenitor cells and their corresponding neuronal subtypes.

## Material and Methods

### Embryos

All animal procedures were approved by the CNRS/Fédération de Recherche de Biologie de Toulouse Animal Experimentation Ethics Committee (C2EA-01) under the protocol number 01024–01.7. The study was carried out in compliance with the European Policy on Ethics. Fertile hen eggs (from *Gallus gallus*), obtained from a local supplier (SCAL l’Isle Jourdain, France), were incubated at 38°C in a humidified chamber for the appropriate duration to yield embryos of Hamburger and Hamilton stages HH10 and HH20 (days E1.5 and E3.5 of development, respectively). C57Bl6 mouse embryos were isolated at E10 after detection of vaginal plugs.

### Cell culture and mitotracker labeling

The HeLa cell line was purchased from the ATCC and cultured in DMEM (GIBCO) supplemented with 10% FCS (Gibco). The DF1 cell line (UMNSAH/DF-1, ATCC CRL-12203, kind gift of Gaël Orieux, UPMC, Institut de la Vision, Paris, France) was cultured in DMEM supplemented with 5% FCS and 5% chick serum (Gibco).

Mitochondria were labeled by incubation of the cells for 40 min with the Mitotracker Red probe (Invitrogen) at 0.1 μM in culture medium. Cell nuclei were detected by Hoechst (Sigma) post-fixation labeling.

### Electroporation

We used the Mito-DsRed expression vector that encodes DsRed2 protein (Discosomosa sp.) in fusion with the Mitochondrial targeting sequence of the human COX-subunit VIII, placed under the control of the pCMV promoter. *In ovo* electroporations were performed as previously described [29] in E1.5 day-old chicken embryos. Electroporated eggs were incubated at 38°C for 2 days, and E3.5 embryos were processed for immunodetection.

### Antibodies

Mitochondria were labeled by immunofluorescence using a mouse antibody specific for the human ATP-synthase (Molecular Probes) or a mouse antibody cocktail specific for the rodent OXPHOS complexes (MitoProfile Total OXPHOS, Mitosciences), or rabbit antibody specific for the human TOM20 mitochondrial protein (Santa-Cruz). Neural progenitor domains were labeled by immunofluorescence with either anti-Pax6 (Covance) or anti-Olig2 (Upstate Millipore) polyclonal antibodies, and mitotic progenitors with anti-P-H3 epitope antibodies (Upstate Millipore) or MPM2 (Mitotic Protein Monoclonal#2, Upstate Biotechnology), which recognizes phosphorylated epitopes at the onset of mitosis. Antibodies directed against the neuronal beta3-tubulin (Sigma) or JC7 (a gift from J. Covault), were used to identify differentiating neurons. SR101-Phalloidin that labels cortical actin in the whole tissue was used to visualize overall cell morphology and DAPI (Sigma) to label nuclear DNA. For Western blot experiments, antibodies against Actin (Chemicon), HSP60 (LK2, Sigma), OXPHOS (Mitosciences), DRP1 and OPA1 (BD-Biosciences), and MFN2 (Abnova) proteins were used.

Primary antibodies were detected using either fluorochrome-tagged secondary antibodies coupled to Alexa 488, 596 or 647 for immunochemistry or HRP-conjugated secondary antibodies for Western blot experiments (Abcam).

### Immunochemistry

Embryos were dissected and fixed for 3 h (chick) or 12 h (mouse) in a 3.7% v/v formaldehyde/PBS solution. Briefly, floating 50-μm vibratome sections were immunostained by successive incubations with the primary antibodies overnight at 4°C, the secondary antibodies for 4 h at room temperature, and rinsing steps. Images were acquired on confocal SP2 or SP8 microscopes (Leica) using X20, X40, and X63 oil objectives.

For immunofluorescence analysis, HeLa and DF1 cells were seeded on glass coverslips and cultured until reaching approximately 80% confluence. After fixation in a 3.7% v/v formaldehyde-PBS solution for 20 min and permeabilization in a 0.5% v/v Triton-X100-PBS solution for 10 min, cells were processed for OXPHOS labeling as described [24].

### Western blot

Protein extracts were prepared from E1.5 and E3.5 chick neural tubes and from HeLa and DF1 cultured cells. Tissue and cell lysis was performed in 50 mM Tris pH 7.5, 5 mM EDTA, 5 mM EGTA, 300 mM NaCl, 1% NP40, 1 mM PMSF lysis buffer, and 100-μg samples of clarified protein lysates were submitted to SDS-PAGE. Immunodetection of Actin, HSP60, OXPHOS, DRP1, OPA1 and MFN2 proteins was performed as previously described [24].

## Results

### Antibodies directed against mitochondrial respiratory complexes allow detecting mitochondria in the chick embryo

**Fig 1:**
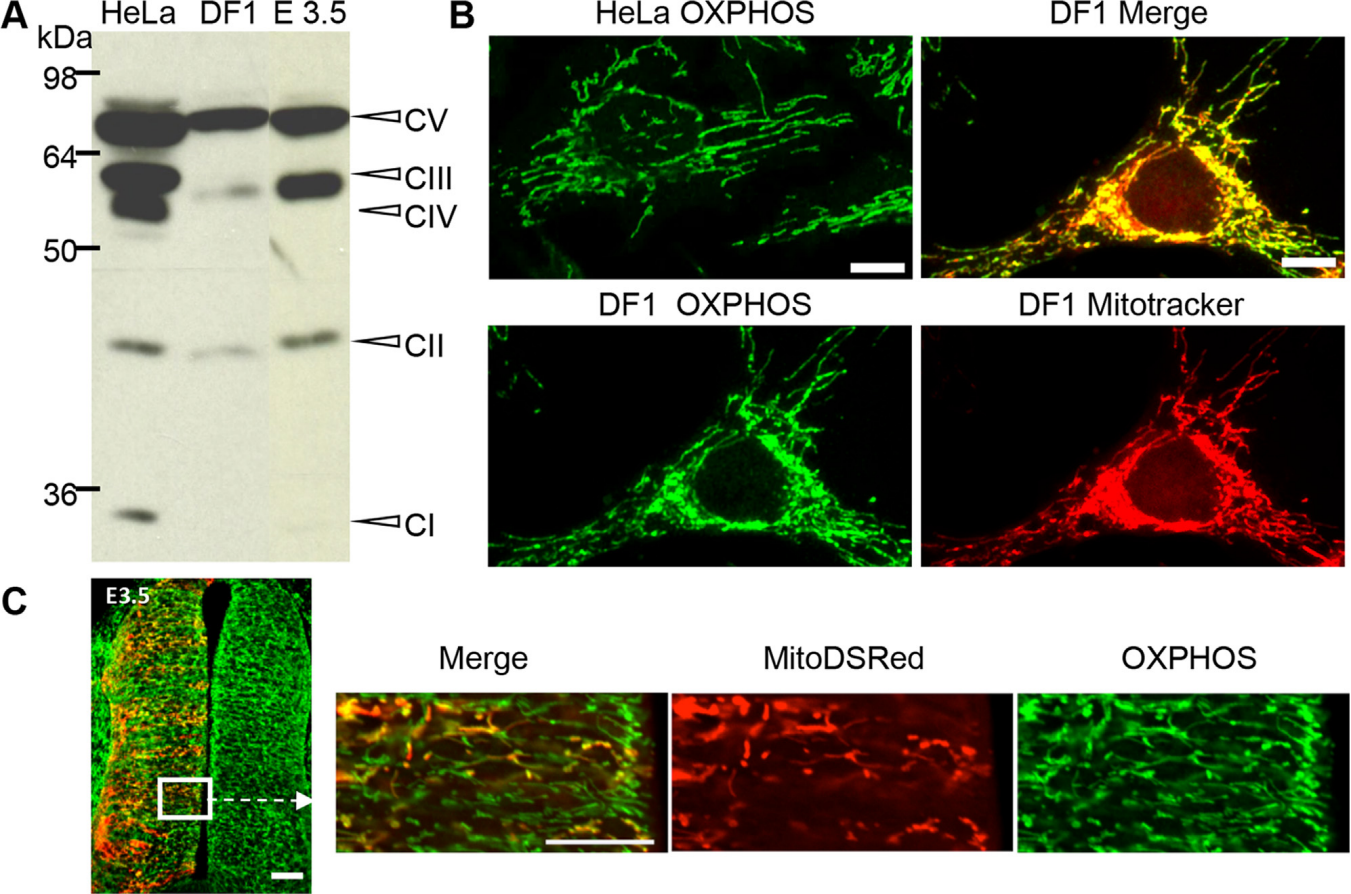
The OXPHOS antibody labels respiratory complex proteins in Western blots and immunocytochemical experiments. (A) Representative immunoblot of OXPHOS antibody labeling, showing the expected 5 proteins in HeLa cells extracts, and proteins from the V, III, and II complexes in chicken DF1 fibroblasts. (B) Fluorescent micrographs of OXPHOS labeling showing green filamentous mitochondria in HeLa cells (upper left panel) and chick DF1 fibroblasts (lower left panel). MitoTracker staining of DF1 cells depicts a red mitochondrial network (lower right panel) superimposed on the green OXPHOS staining (upper right panel) (scale bar 10 μm). (C) Confocal micrograph of a transversal section from a E3.5 chicken neural tube showing OXPHOS-immunostained mitochondria (green) and a mosaic staining of Mito-DsRed-labeled mitochondria on the left side (red) (scale bar 50 μm). Higher magnification at the level of the ventricular zone illustrates the co-localization of the Mito-DsRed (red) and OXPHOS staining (green) (scale bar, 20 μm).

In order to visualize mitochondria in the chick neural tube, we used the OXPHOS antibody cocktail directed against one subunit of each of the 5 mitochondrial mammalian respiratory complexes. We first verified the cross-reactivity of this antibody by Western blot using chicken-derived fibroblasts (DF1 cells) as well as neural tubes dissected from E3.5 embryos. The expected proteins from the 5 complexes were detected in human epithelial HeLa cell extracts, but only those from complexes V, III, and II were clearly visualized in both DF1 cells and neural tubes extracts (Fig 1A). We next compared the signal obtained by immunocytochemistry in HeLa and in DF1 cells (Fig 1B). As expected, the OXPHOS antibody cocktail labeled a filamentous mitochondrial network in HeLa cells [30]. In DF1 cells, the OXPHOS signal was clearly similar to the MitoTracker staining, which specifically labeled filamentous mitochondria (Fig 1B). Finally, we electroporated the mitochondria-targeted fluorescent protein-encoding vector Mito-DsRed in E1.5 embryos and performed immunodetection with the OXPHOS antibody cocktail at E3.5. Fig 1C shows that OXPHOS staining extensively co-localized with the red “mosaic” signal indicative of mitochondria in electroporated cells. Altogether, these results indicated that the OXPHOS antibody cocktail was suitable for analyzing mitochondrial morphology in the chicken neural tube.

### Mitochondrial morphology changes during embryonic neurogenesis in the chicken neural tube

To compare the mitochondrial morphology in neural progenitors versus differentiating neurons, we used E3.5 embryos because numerous proliferating neural progenitors are still present in the ventricular zone, while differentiating neurons accumulate in the mantle zone. To precisely analyze the changes occurring in the mitochondrial network related to cell differentiation, we focused on progenitors of motor neurons (pMNs) and their corresponding differentiating motor neurons (MNs), easily traceable with specific markers and confined to the ventral part of the spinal cord. The pMNs were identified using an antibody directed against the transcription factor Olig2. Differentiating MNs were labeled using the BEN/SC1/DM-GRASP antibody (named JC7), which recognizes a glycoprotein of the immunoglobulin superfamily on motor neurons and on sensory neurons located outside the spinal cord. We also used phalloidin to detect F-actin and to visualize the morphology of the cell.

**Fig 2:**
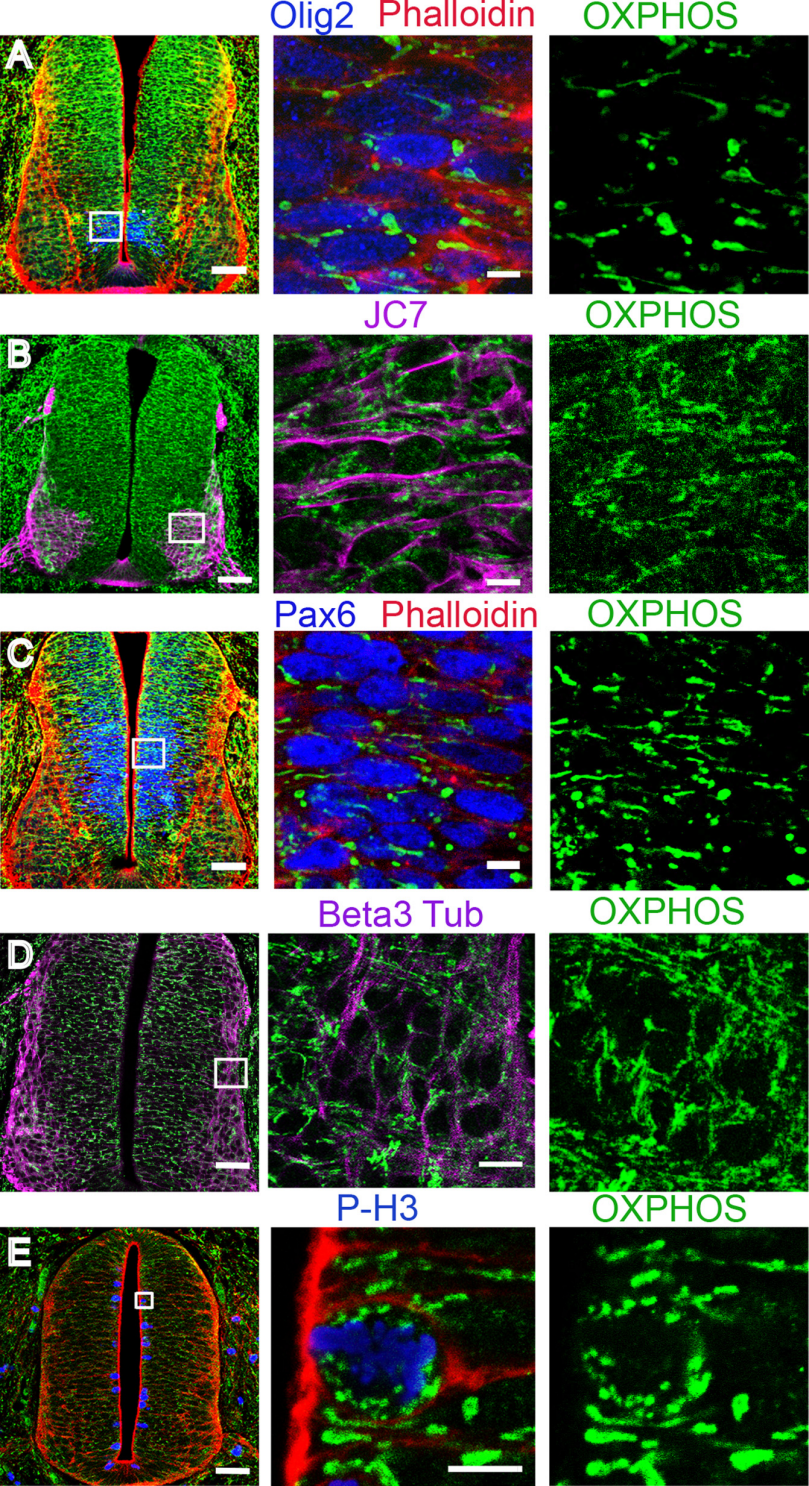
Mitochondrial reshaping accompanies neuronal differentiation in the chicken neural tube. Panels A through E represent confocal micrographs of green OXPHOS-stained mitochondria in E3.5 transversal sections (scale bar 50 μm), magnified in the mid- and right-side panels (scale bar 10 μm). In panels A, C, and E, SR101-Phalloidin signaling (red) was used to mark the cellular contours. Neuronal progenitors, immunolabeled either with Olig2 for future motor neurons (A, blue) or Pax6 for future interneurons (C, blue), contain short and thick mitochondria. In panel B, JC7-immunodetection (purple) delineates the surface of differentiating motor neurons endowed with a longer and denser mitochondrial network. In panel D, the mitochondrial network of beta3-tubulin-stained differentiating neurons (purple) is also long and dense. In panel E, P-H3-immunostained (blue) mitotic progenitors are shown to contain very small and round mitochondria.

**Fig 3:**
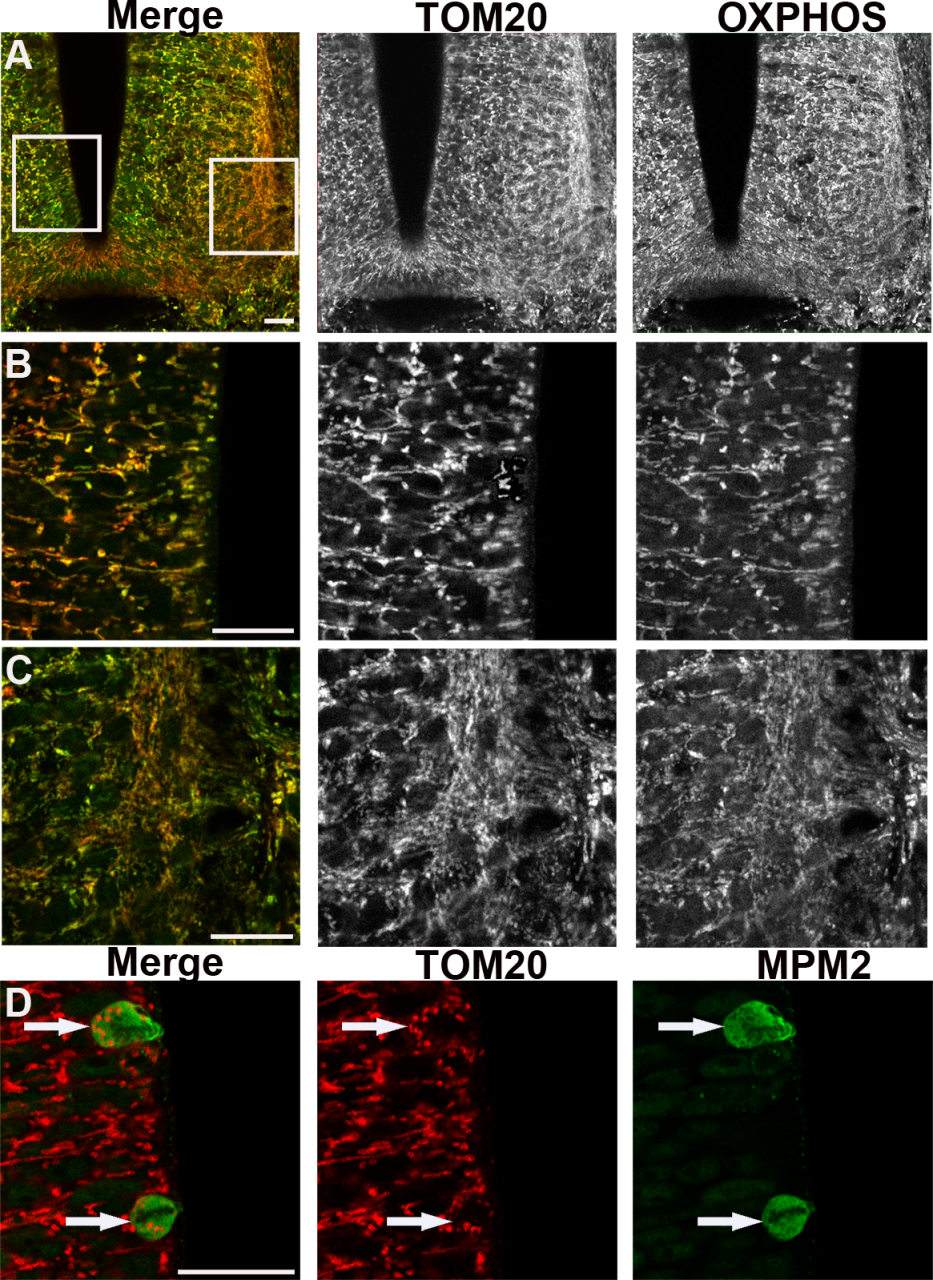
Immunodetection of the outer mitochondrial membranes TOM20 reveals the same changes in mitochondrial morphology than OXPHOS. Panels A to C represent confocal micrographs of TOM20 (red) and OXPHOS (green) mitochondria staining in cross-sections of the spinal cord of E3.5 chick embryo. Panel A is a low magnification indicative of the enlargement positions shown in B (left) and C (right). In panel B neural progenitors display short and thick labeled-mitochondria (black and white). In panel C differentiating neurons show a longer and denser mitochondrial network (red or green). Note the evident overlay of the two red and green stainings (left panels). Scale bars, 20μm. Panel D illustrates the TOM20-labeled very small and round mitochondria (red) observed in mitotic cells (white arrows) stained with MPM2 antibody (green). Scale bars, 20μm.

OXPHOS immunodetection in pMNs and differentiating MNs revealed a clear difference in mitochondrial morphology. In pMNs, mitochondria appeared as short and thick well-delineated elements (Fig 2A), distinctly detected around the nucleus. In differentiating MNs, mitochondria were unambiguously thinner, elongated, and organized into a more complex and denser network (Fig 2B). The same observations were made in the population of spinal progenitors expressing the transcription factor Pax6 fated to differentiate into interneurons (Fig 2C) and in differentiating neurons visualized using the pan-neuronal marker beta3-tubulin (Fig 2D). We confirmed these observations with an antibody directed against another mitochondrial protein, TOM20, located in the outer mitochondrial membrane, which gives a signal fully superimposable with OXPHOS staining (Fig 3A–3C). Altogether, these data indicate that mitochondrial morphology changes at the transition from neural progenitors to differentiating neurons and that this shape modification is a generic event associated with neurogenesis rather than specific to a subtype of neurons.

In the proliferating progenitor population, cells are either in interphase or in mitosis, dividing into two daughter cells. We did not detect any difference in interphasic cells suggestive of mitochondrial rearrangements within G1, G2 and S phases. However, in mitotic cells, located apically and identified using P-H3 or MPM2, the morphology of mitochondria was strikingly different than in interphase progenitors as shown using either OXPHOS (Fig 2E) or TOM20 staining (Fig 3D). They appeared as very small and round dots distributed around the condensed chromosomes. This indicates a drastic reshaping of the mitochondrial network occurring in mitosis.

### Mitochondrial morphology changes during neurogenesis in the mouse neural tube

**Fig 4:**
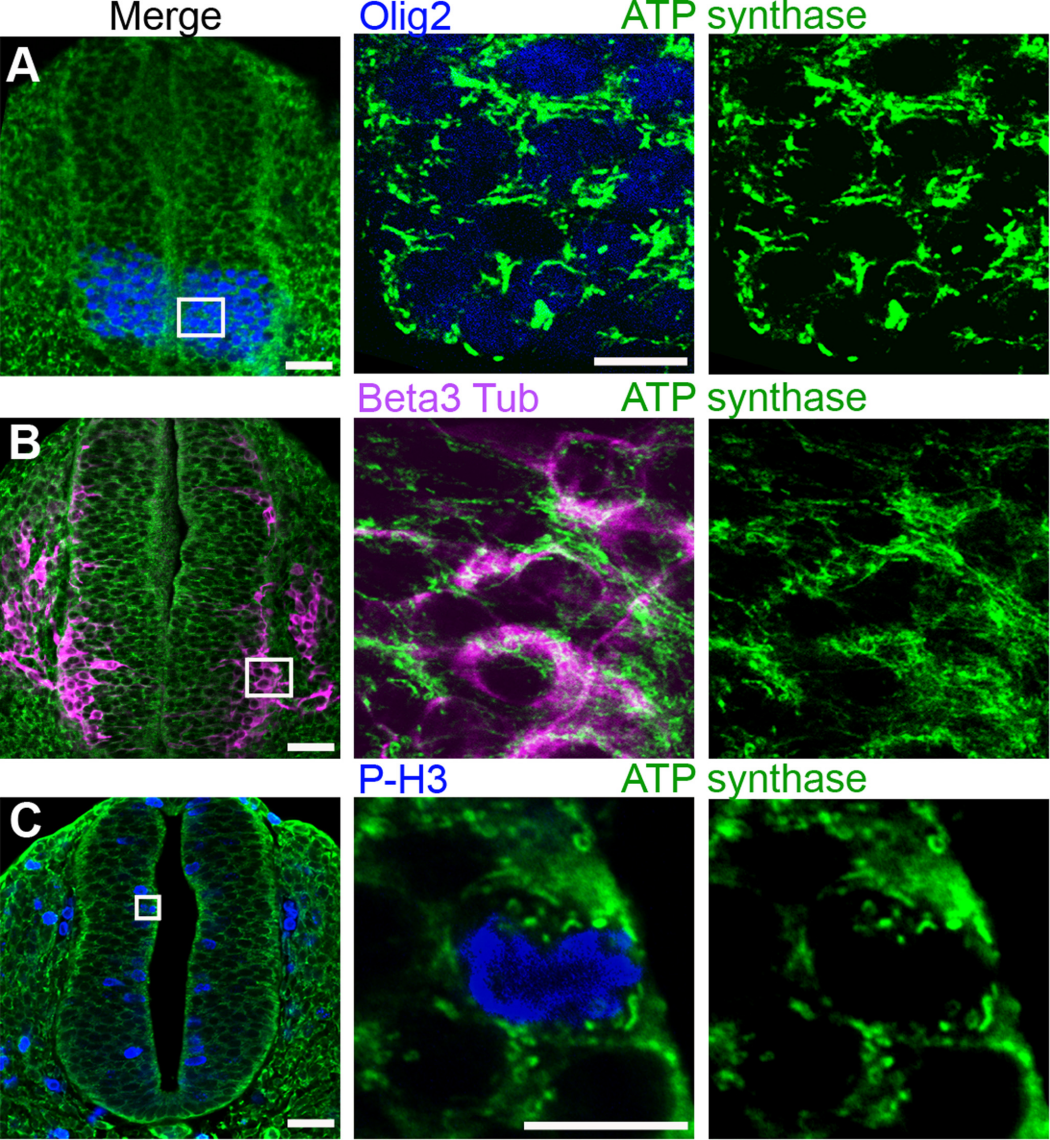
Mitochondrial reshaping accompanies neural progenitor differentiation in the mouse neural tube. Panels A to C represent confocal micrographs of green ATP synthase-stained mitochondria in E9.5–10 neural tube transverse sections (scale bar 50 μm), magnified in the mid- and right-side panels (scale bar 10 μm). In panel A, Olig2-stained neuronal progenitors (blue), future motor neurons, contain short and thick mitochondria. In panel B, beta3-tubulin-stained (purple) differentiating neurons are endowed with a longer and denser mitochondrial network. In panel C, P-H3-stained (blue) mitotic progenitors are shown to contain very small and round mitochondria.

To broaden our findings, we verified if the different mitochondrial morphological characteristics observed in the chicken neural tube were also observed in the developing mouse embryo. We thus used an antibody specific for the 5^th^ mitochondrial respiratory complex, ATP-synthase, which specifically labels mitochondria (S1 Fig) and appeared more suitable than the whole OXPHOS cocktail for this tissue in terms of signal/noise ratio. We analyzed mouse embryos at the E10 development stage, which corresponds to the E3.5 stage in chicken embryos. As in the chick neural tube, short and thick mitochondria were observed in the mouse neuroepithelium along the whole ventro-dorsal axis of the neural tube, including Olig2-labeled subdomains (Fig 4A). Conversely, in the mantle zone, the beta3-tubulin-labelled differentiating cells contained elongated and branched mitochondria (Fig 4B), in the population of spinal MNs as well as in inter-neurons (data not shown). Very small and round mitochondria were again observed in the P-H3-labelled mitotic progenitors (Fig 4C). These observations indicate that this change of shape accompanying spinal neurogenesis is conserved between chick and mice species, suggesting that it is a common property of vertebrate neural cells.

### A progressive rearrangement of the mitochondrial network accompanies neural progenitors maturation

**Fig 5:**
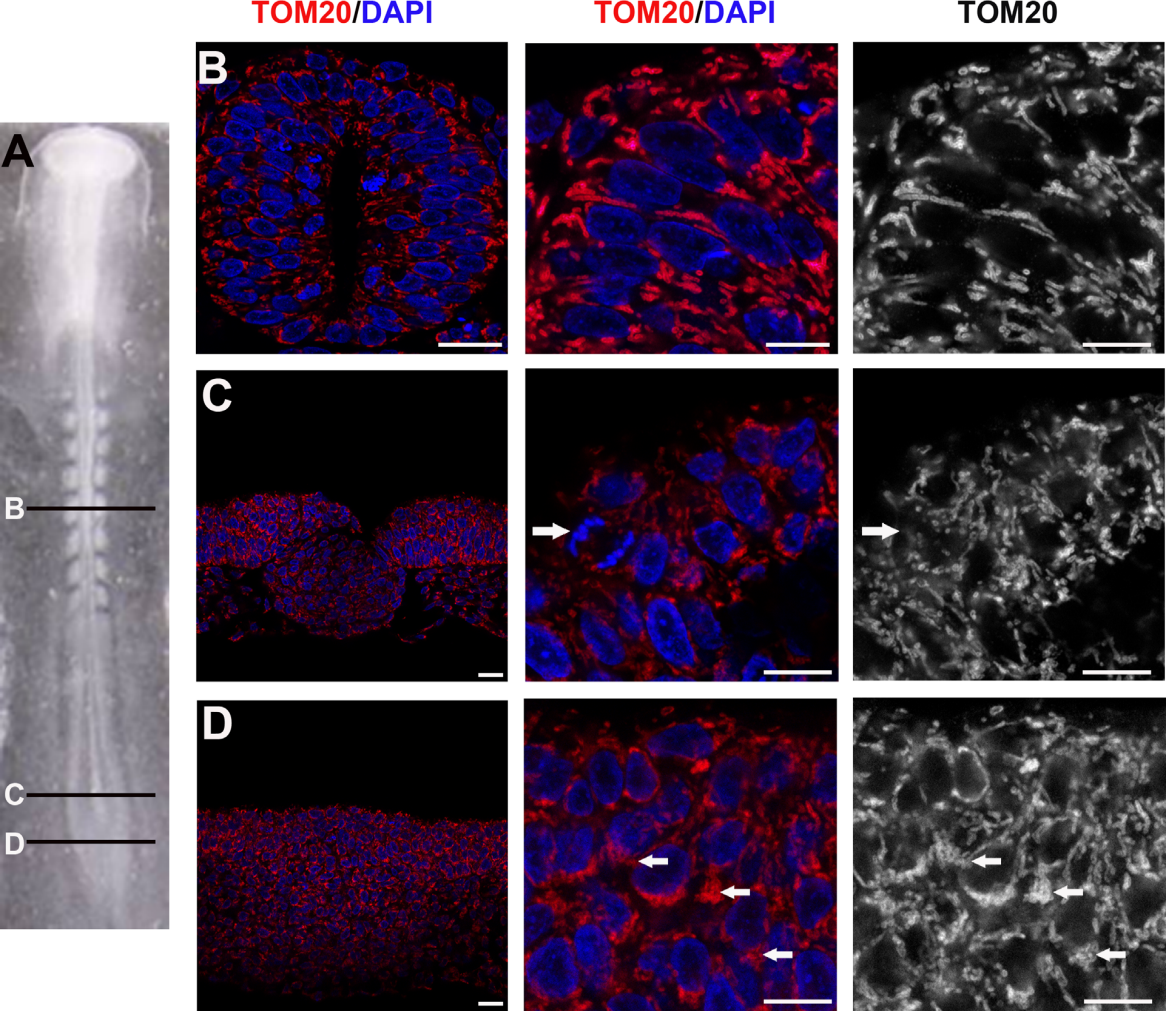
Rearrangement of mitochondria in maturating neural progenitors. (A) Dorsal view of E1.5 (stage-10HH) chick embryo; (B-D) cross-sections of panel A. Images in the center and right-hand lanes are enlargements from images in the left lane. (B) TOM20 (red or white) and DAPI (blue) show the well delineated and organized mitochondrial network around the nuclei in the neural tube (Scale bars, 20μm left and mid panels; 10μm right panel). Panels C and D are confocal micrographs of TOM20-stained mitochondria (red and white) and DNA (DAPI, blue) in younger progenitors located respectively in the caudal neural plate (C) and in the caudal stem zone (D). Arrows in B and C point to a mitosis and arrows in D point highly packed mitochondria (Scale bars, 20μm left panel; 10μm, mid and right panels).

Our observations underlined a morphological change in mitochondria morphology accompanying the transition from neural progenitors to differentiating neurons. To complete our data, we then asked if mitochondrial morphology was conserved during the main temporal steps of neural progenitors maturation. We thus stained E1.5 embryos with TOM20 antibodies and analyzed mitochondrial shape at different rostro-caudal levels: in the caudal stem zone containing uncommitted progenitors (Fig 5A and 5D); in the caudal neural plate where cells are committed to the neural fate (Fig 5A and 5C) and in the more mature young neural tube (Fig 5A and 5B). As observed at E3.5, proliferating progenitors from all these rostro-caudal levels at E1.5 also display numerous short and thick mitochondria (5A–5D). The main difference noticed is a re-arrangement of these thick mitochondria within the cells as progenitors become more mature. Indeed, in the caudal stem zone, mitochondria appeared tightly packed (Fig 5D), while displaying a more organized and delineated network in the neural tube (Fig 5B); an intermediate situation was observed at the level of the caudal neural plate (Fig 5C). Together with the experiments made at E3.5, these observations indicate that thick and short mitochondrial morphology is a general feature of neural progenitors, and that mitochondria rearrange concomitantly with the maturation of progenitors in the developing neural tube.

### Changes in mitochondrial morphology are not related to major changes in fission and fusion protein levels

**Fig 6:**
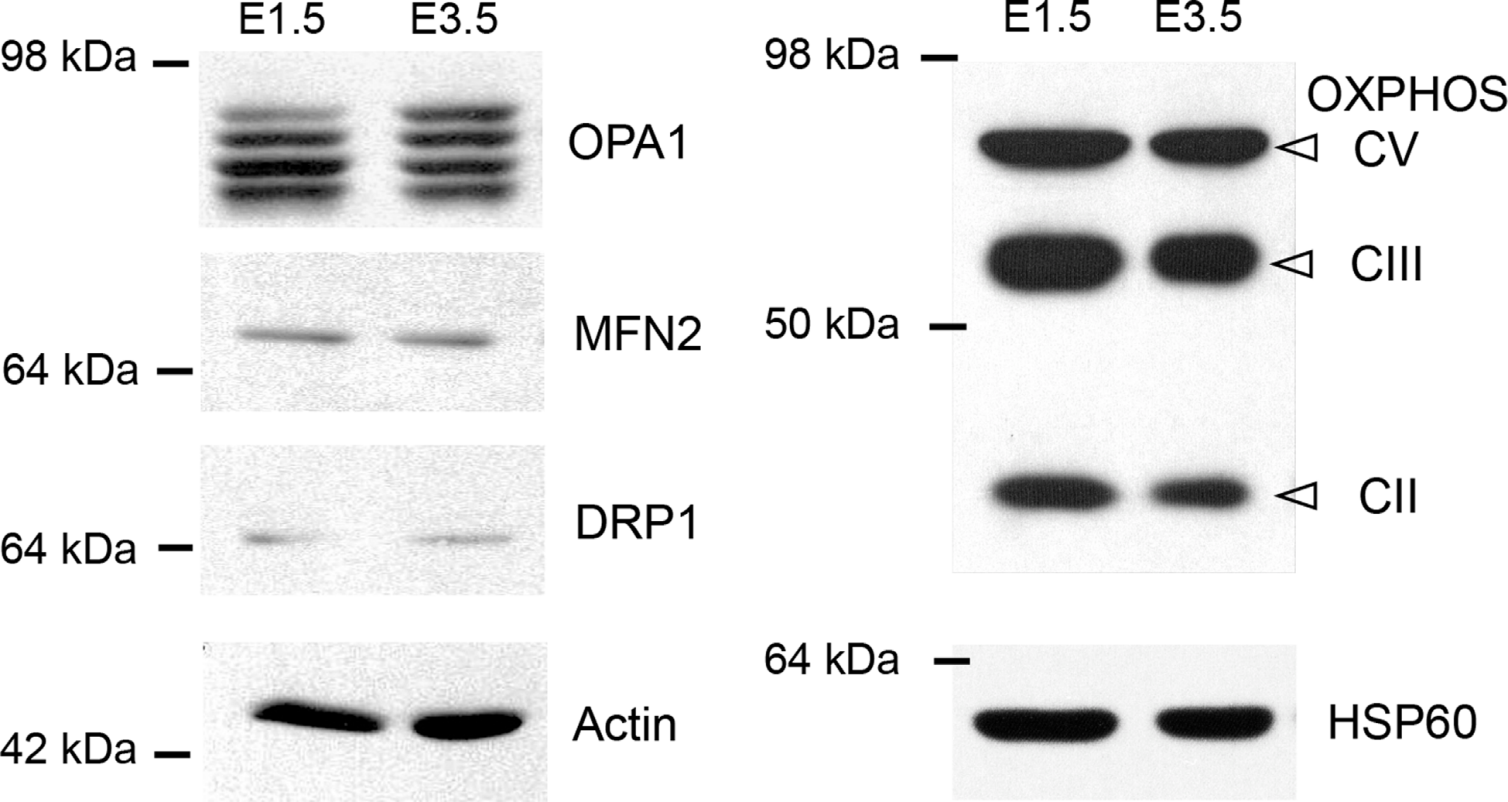
Differential expression of effectors of mitochondrial dynamics in E1.5 and E3.5 chicken neural tube extracts. Representative immunoblot showing the expression of the mitochondrial dynamics proteins, DRP1, MFN2 and OPA1, as well as mitochondrial respiratory complexes V, III and II and HSP60 protein in E1.5 and E3.5 chicken neural tubes extracts, with actin used as control. While DRP1 and MFN2 are represented as single bands of similar intensities at both ages, staining of the OPA1 isoforms reveals an increase in the longest isoform in the E3.5 extract compared with the E1.5 extract.

Changes in mitochondrial morphology could be due to a differential expression of the proteins that mediate fission and fusion. We therefore compared the quantities of the fusogenic MFN2 and OPA1 proteins and of the fission protein DRP1 in E1.5 chick neural tubes, containing mostly cycling neural progenitors, with those in E3.5 neural tubes, which contained numerous neurons, using Western blotting (Fig 6). At both stages, MFN2 and DRP1 proteins appeared as single bands, while OPA1 staining displayed the classical isoforms (Fig 6) [30]. The only difference we observed is an approximately 1.5-fold increase in the expression of the longest isoform of OPA1 (159 ± 31%); however, the difference did not reach statistical significance (p = 0.063). At both stages, labeling with two specific mitochondrial antibodies gave the same pattern (Fig 6). No difference of OXPHOS and HSP60 levels was detected, suggesting that mitochondrial biomass and respiratory complexes quantities increased to the same extent than the total protein amount and actin between E1.5 and E3.5.

## Discussion

Here, we investigated the morphology and distribution of mitochondria in the neural tube of E1,5, E3.5 chicken and E10 mouse embryos upon neurogenesis. We report the first *in vivo* evidence that mitochondria undergo morphological reshaping correlating with the transition from proliferating neural progenitors to differentiating neurons. In the proliferating ventricular zone, mitochondria in the mitotic cells lying at the apical side were very small and round, while they appeared short and thick in interphase cells. In contrast, in differentiating neurons, mitochondria formed a thin and dense network. This reorganization of the mitochondrial network is not specific of a subtype of progenitors or neurons, suggesting that this is a general event accompanying neurogenesis in the spinal cord.

In agreement with previously reported *in vitro* studies, we observed that mitochondrial morphology in neural progenitors varied between interphase (G1/S/G2 phases) and mitosis. Mitochondrial fission has indeed been reported to be increased during mitosis through the activating phosphorylation of DRP1 by the mitotic kinase CDK1/cyclin B [31], while the recovery of longer mitochondria when exiting mitosis was regulated by the anaphase-promoting complex-dependent DRP1 ubiquitination and degradation [32].

Neural progenitors contain short and thick mitochondria, which progressively display a more delineated morphology with neuroepithelium maturation. The low complexity of the mitochondrial network in the neuroepithelium is reminiscent of what was described for many pluripotent cell types *in vitro* [33]. These cells contain rare, short and small perinuclear mitochondria and are characterized by a glycolytic metabolism that limits ROS production and oxidative damage [3,4]. Accordingly, reactivation of glycolysis, which is an obligatory step for adult cells reprograming into iPSCs, occurs with mitochondrial shortening and degradation [3]. Thus, the low complexity of the mitochondrial network that we observed *in vivo* in the neuroepithelium could be related to a glycolytic state.

Upon neurogenesis, progenitors exit the cell cycle, delaminate basally and then migrate into the mantle zone, where they start to develop neuritic processes. We showed that this transition from proliferating progenitors to differentiated neurons is accompanied by an increase in mitochondrial length and network density. This morphological transition could be related to an increased expression of the long OPA1 fusogenic isoform [34] in differentiating cells of E3.5 chicken neural tubes, as well as to post-translational modifications known to regulate the function of mitochondrial dynamics actors but undetectable in our experiment [35] So, as previously demonstrated *in vitro*, neuronal differentiation *in vivo* seems to occur with mitochondrial biogenesis and fusion, altogether evocative of a metabolic shift from glycolysis to oxidative phosphorylation [4,5,11,36]. As previously described [37,38] this mitochondrial filamentation could allow mitochondria to reprogram into more efficient ATP providers able to fulfill the high energy demands of differentiating cells. Newborn neurons could thus be energized to engage in axonogenesis and competition for neurotrophic factors during neuronal growth [39,40].

To conclude, our data shed new light on the various changes occurring in the mitochondrial network along with spinal neurogenesis. These findings suggest that mitochondrial dynamics could play a role in the neurogenic process. Interestingly, a possible causal link between this process and molecular pathways controlling embryonic stem cell differentiation was recently reported. Experimental ablation of OPA1/MFN2 fusogenic proteins was indeed shown to impair differentiation of ESC into cardiomyocytes *via* calcineurin and Notch signaling [11]. Furthermore, Notch signaling was recently shown to control both glycolysis and mitochondrial activity [41,42]. Thus, during neural tube development, mitochondrial dynamics could contribute to, and act with, the Notch pathway [43], well-known to control the balance between progenitor maintenance and differentiation in the developing nervous system [44]. Beyond the nucleus-driven decisions, mitochondrial differentiation could thus pave the way for growth/differentiation factor-dependent processes.

## Supporting Information

S1 Fig ATP synthase immunodetection in mouse neurons extracts and in fixed neurons. (TIFF)
