# Supplementary figures and images for: Mitochondrial Reshaping Accompanies Neural Differentiation in the Developing Spinal Cord
Source: PLoS One. 2015 May 28;10(5):e0128130. doi: 10.1371/journal.pone.0128130 (PMC4447341; doi:10.1371/journal.pone.0128130)

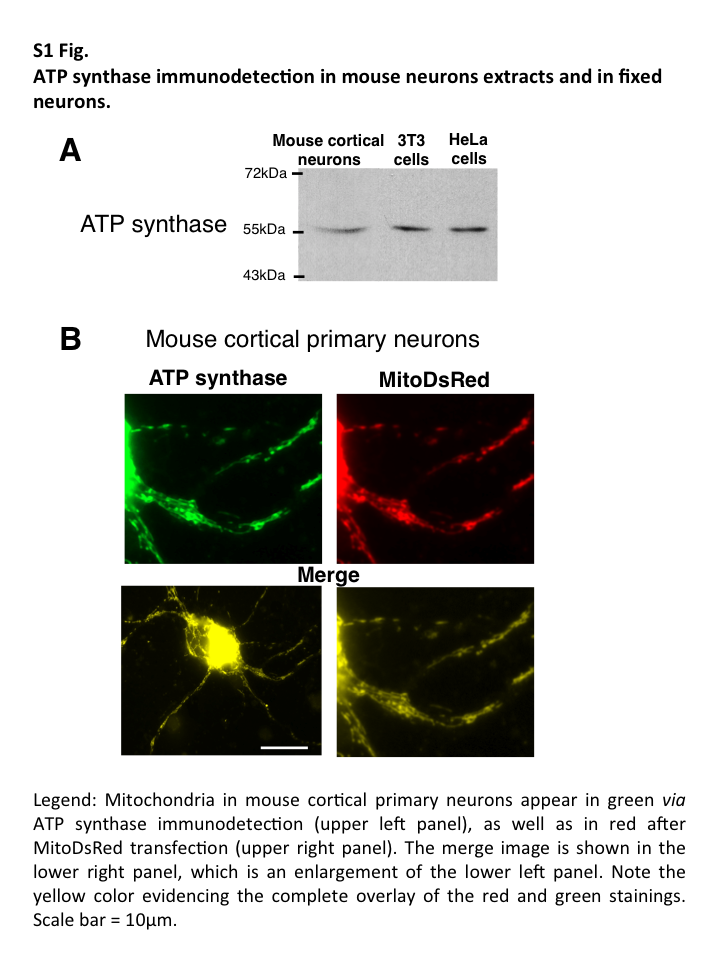

Supplement: S1 Fig — (TIFF) [file pone.0128130.s001.tiff]
